# iTRAQ-based analysis of sperm proteome from normozoospermic men achieving the rescue-ICSI pregnancy after the IVF failure

**DOI:** 10.1186/s12014-018-9203-3

**Published:** 2018-08-21

**Authors:** Xin Liu, Gensheng Liu, Juan Liu, Peng Zhu, Jiahui Wang, Yanwei Wang, Wenting Wang, Ning Li, Xuebo Wang, Chenglin Zhang, Xiaofang Shen, Fujun Liu

**Affiliations:** 1 Central Laboratory, The Affiliated Yantai Yuhuangding Hospital of Qingdao University, Yantai, 264000 Shandong People’s Republic of China; 2 Reproductive Center, Tianjin Aiwei Hospital, Tianjin, 300011 People’s Republic of China; 3 Department of Clinical Laboratory, The Affiliated Yantai Yuhuangding Hospital of Qingdao University, Yantai, 264000 Shandong People’s Republic of China; 4 Reproductive Center, Beijing BaoDao Obstetrics and Gynecology Hospital, Beijing, 100000 People’s Republic of China

**Keywords:** Male unexplained infertility, Sperm proteins, iTRAQ, IVF pregnancy, R-ICSI pregnancy

## Abstract

**Background:**

In the assisted reproduction, the infertile molecules of spermatozoa from normozoospermic men who underwent the unexplained failure of in vitro fertilization (IVF) due to the lack of sperm binding to the normal zona pellucida, and then achieved pregnancy with the rescue intracytoplasmic sperm injection (R-ICSI) remain unclear. More works are still necessary to explore this male infertile mechanism.

**Methods:**

Normozoospermicmen with the IVF pregnancy and normozoospermic men with the R-ICSI pregnancy after the conventional IVF failure were collected. iTRAQ-based proteomic approach were performed to reveal the new infertile causes between the IVF pregnancy men and the R-ICSI pregnancy men. To validate the confidence of proteome data, the individual samples were analyzed by western blot and immunofluorescence. Further, the spontaneous acrosome reactions were measured to evaluate the sperm quality.

**Results:**

Compared with IVF pregnancy group, 56 sperm proteins were differentially expressed in the R-ICSI pregnancy group. Bioinformatic analyses (PANTHER, DAVID, PubMed and STRING) indicated these altered sperm proteins were involved in various molecular functions: reproduction, chromosome organization, and sperm-oocyte interaction. Moreover, the confidence of proteome data was confirmed by western blot and immunofluorescence using the individual samples, which were consistent with our proteomic data. Additionally, an increased rate of the spontaneous acrosome reaction rate was found in the R-ICSI pregnancy group.

**Conclusions:**

The sealtered sperm proteins and the increased spontaneous acrosome reaction rate might account for this unexplained male infertility in the R-ICSI pregnancy patients. The present proteomic results will throw light on the better understanding of the unexplained infertile mechanisms underlying these normozoospermic man who achieved R-ICSI pregnancy after IVF failure.

**Electronic supplementary material:**

The online version of this article (10.1186/s12014-018-9203-3) contains supplementary material, which is available to authorized users.

## Background

Approximate 15% of couples are suffered from infertility, and male factors contribute to 50% of infertile cases. Male infertility is usually characterized with abnormal sperm motility, concentration, and morphology, which is respectively defined as asthenozoospermia, oligozoospermia, and teratozoospermia. Although the etiology of male infertility is still unclear, ICSI is an effective treatment for man with severe infertility. Additionally, IVF is also used for men with normal semen parameters. In the conventional IVF, a few normozoospermic men experience unexplained fertilization failures with a significant decrease of sperm binding to the zona pellucid [1, 2]. Considering the normal maturity, number, and quality of retrieved oocytes, male factors might be the major infertile causes, which has been called ‘hidden’ male factor infertility [3].

A few clinical outcomes indicate the conventional assessment of semen quality cannot satisfy the requirements for the diagnosis of sperm dysfunction [4, 5]. In order to disclose the unexplained male infertility, proteomic technology has been performed to define the potential sperm proteins in male reproduction. In 2004, two-dimensional electrophoresis has been used to explore the altered sperm protein from a patient experienced failed fertilization during IVF treatment. Compared with the fertile donors, two up-regulated sperm proteins are identified as prolactin-inducible protein and outer dense fiber protein 2 [6]. Anotherproteomic analysis of spermatozoa from patients with no sperm bound to the zona pellucida has been carried out by two-dimensional fluorescence difference gel electrophoresis. Seventeen differentially expressed sperm proteins are identified by mass spectrometry, which might provide the potential biomarkers for the diagnosis and prognosis of the IVF-failed infertility [7]. LC–MS method has also been applied to reveal the differential sperm proteins in the IVF-failure group versus the fertile group. A list of sperm proteins may provide the new understanding of the mechanisms of the IVF failed patients [8].

The present study aimed to deeply reveal sperm protein defects associated with failed fertilization at conventional IVF. The normozoospermic man who achieved fertilization by the IVF or R-ICSI treatment were included, and all the subsequent pregnancies of their female partners were confirmed by an ultrasound detection of fetal heartbeat 6 weeks after embryo. We performed the quantitative proteomic analysis using iTRAQ labeling and LC-MALDI mass spectrometry to disclose differential sperm proteins between the IVF pregnancy group and the R-ICSI pregnancy group. All findings will provide the potential biomarkers for the diagnosis of male infertility and facilitate the better understanding of male reproductive processes.

## Methods

### Patients enrollment and sperm sample preparation

According to World Health Organization (WHO) guidelines (WHO Laboratory Manual for the Examination and Processing of Human Semen, 5th) [9], the semen samples included in this study were from 20 normozoospermic men with IVF pregnancy and 20 normozoospermic men with R-ICSI pregnancy. Their female partners met the following criteria: aged no more than 38 years, normal body mass index (BMI) (18.5 ≤ BMI ≤ 23.9 kg/m^2^) [10–12], without chromosomal abnormalities, endocrine disease, endometriosis or polycystic ovary syndrome (PCOS). Ovulation stimulation were with the long protocol in their first fresh egg retrieval cycle. After IVF or R-ICSI treatment, an ultrasound detection of fetal heartbeat 6 weeks after embryo was used to confirm their pregnancy. After 30 min of liquefaction, the semen samples were centrifuged at 300×*g* for 20 min to separate spermatozoa from seminal plasma in a 45–90% discontinuous SpermGrad (Vitrolife, Göteborg, Sweden) gradient. After washing twice in phosphate-buffered saline, the pellet was collected by 450×*g* centrifugation for 10 min. The proteins were solubilized in 8 M urea and protease Inhibitor Cocktail (Roche Diagnostics, Oslo, Norway). After centrifugation (20,000×*g*, 30 min, 4 °C) [13, 14], the protein supernatant was collected and its concentration was determined by the Bradford method [15]. This work has been approved by the Ethics Committee of Beijing BaoDao Obstetrics and Gynecology Hospital, and written informed consents were obtained from all participants.

### Spontaneous acrosome reaction measurement

According to the previous method, spontaneous acrosome reaction was assessedby using FITC-PSA (Sigma-Aldrich, Steinheim, Germany). Briefly, the above resultant sperm pellet was placed on gelatin-coated slides, and fixed with methanol for 10 min. After incubation with FITC-PSA at room temperature in the dark for 30 min, propidium iodide counterstaining visualized the sperm nuclei. More than 200 cells on each section were examined to calculate the percentage of acrosome-reacted sperm under a confocal laser scanning microscope (LSM-510 META; Carl Zeiss, Jena, Germany) [16–18].

### iTRAQ analysis of sperm samples

Equal amounts of sperm proteins from 20 normozoospermic men of IVF pregnancy group, and 20 normozoospermic men of R-ICSI pregnancy group were pooled, and the resultant samples from the IVF or R-ICSI pregnancy group were respectively separated into 2 tubes (100 μg each) with the pH adjusted to 8.5. Furtherly, the samples were treated with 20 mM dithiothreitol (DTT) at 56 °C for 60 min, and 50 mM iodoacetamide (IAA) in the dark for 30 min. After the samples were digested by trypsin (sequencing grade, Promega, France), the tryptic peptides were labelled by iTRAQ reagents, and then dried by a SPD2010 SpeedVac concentrator system (Thermo, North Carolina, USA) [19].

## 1st pH 10.0 RPLC separation

The first dimension RP separation was carried out with a Durashell RP column (5 µm, 150 Å, 250 mm × 4.6 mm i.d., Agela, Tianjin, China) on the PF-2D HPLC System (BeckmanCoulter, Fullerton, California, USA). A gradient elution was achieved by mobile phase A (2% acetonitrile, adjusted pH to 10.0 using NH_3_·H_2_0) and B (98% acetonitrile, adjusted pH to 10.0 using NH_3_·H_2_0) at an eluent flow rate of 0.8 ml/min [20]. Twenty fractions of above labelled peptides were collected and dried by a SPD2010 SpeedVac concentrator system (Thermo, North Carolina, USA).

## 2nd pH 3.0 RPLC coupled with MALDI Mass Spectrometry

Briefly, a linear gradient formed by buffer A (2% acetonitrile, 0.1% formic acid) and buffer B (98% acetonitrile, 0.1% formic acid) was used to separate the above fractions at a flow rate of 0.5 μL/min. Then the eluted peptides were mixed with matrix solution (5 mg/mL in 70% acetonitrile, 0.1% trifluoroacetic acid) pushed by additional syringe pump, and spotted on the MALDI plate (AB SCIEX, Massachusetts, USA) using the Tempo™ LC-MALDI Spotting System (AB SCIEX, California, USA). The 616 spots on each plate were analyzed by a 5800 MALDI-TOF/TOF mass spectrometry (AB SCIEX, Massachusetts, USA). The top 40 ions of the full-scan MS (m/z range from 800 to 4000) were chosen for further tandem MS/MS sequencing [19].

### Identification and quantification of sperm proteins

The identification of sperm protein was done with the ProteinPilot™ software (version 4.0.1; AB SCIEX). A reviewed UniProtKB/Swiss-Prot database (2017_12 released human database, 20,243 entries) was used to search the tandem MS/MS spectrum, and a decoy database (programmed in the ProteinPilot™software) was applied to control the data quality (FDR < 0.01). The searching parameters were iTRAQ 4plex mode, trypsin enzyme, carbamidomethyl cysteine, maximum allowed missed cleavages 1, and biological modifications programmed in the algorithm. Protein abundances were average ratios of all quantified peptides calculated by the areas of the monoisotopic peaks. The average ratio of four pairs (116:114, 117:114, 116:115, and 117:115) in two repeat experiments was used to determine the statistical alteration of sperm proteins with a confidence interval of 95% (*P* value < 0.05) [19, 21].

### Bioinformatic analysis

Altered expressed sperm proteins were characterized by the online PANTHER (Protein Analysis THrough Evolutionary Relationships) (released 13.1, 2018-02-03) (http://pantherdb.org/) [22], DAVID (The Database for Annotation, Visualization and Integrated Discovery; released 6.8, 2016-10) (https://david.ncifcrf.gov/) [23], and the literatures from Pubmed (https://www.ncbi.nlm.nih.gov/pubmed). Each protein was classified into only one category. The protein–protein interaction network was established by the STRING (search tool for recurring instances of neighbouring genes; released 10.5, 2017-05-14) (http://string-db.org/) [24].

### Western blot analysis

Fifty μg sperm proteins were separated by the SDS-PAGE gel, and then transferred to the nitrocellulose membrane. After blocking with the skimmed milk, and incubating with the primary antibody (Anti-ZPBP1, ab97691, Abcam, Cambridge, USA; Anti-ACRBP, ab64809, Abcam, Cambridge, USA) and HRP-conjugated secondary antibody (ZDR-5306, Zhong-Shan Biotechnology, Beijing, China), the immune-reactive proteins on membrane were visualized by chemiluminescence reagents (Pierce, Rockford, IL, USA). The bands were scanned with a Z320 scanner (Founder, Beijing, China), and analyzed using the ImageJ software (http://imagej.nih.gov/ij/).

### Immunofluorescence quantification of spermatozoa protein expression

Briefly, the above sperm pellet was placed on gelatin-coated slides, fixed with methanol, and blocked with BSA. After incubating with the primary antibody (Anti-ZPBP1, ab97691, Abcam, Cambridge, USA) and the FITC-labelled secondary antibody (ZF-0311, Zhongshan golden bridge biotechnology, Beijing, China), all sections were mounted with glycerol and determined with a LSM-510 META confocal laser scanning microscope (Carl Zeiss, Jena, Germany). The intensity and percent of spermatozoa with positive Immunofluorescence staining were assessed according to the previous method [25].

### Statistical analysis

The data of two group means in this study were calculated using the unpaired *t* test with *P *< 0.05 of statistical significance. All analysis was performed with software of Statistical Package for the Social Sciences (SPSS v. 18.0, Chicago, IL, USA).

## Results

### Semen parameters of normozoospermic men

**Fig. 1:**
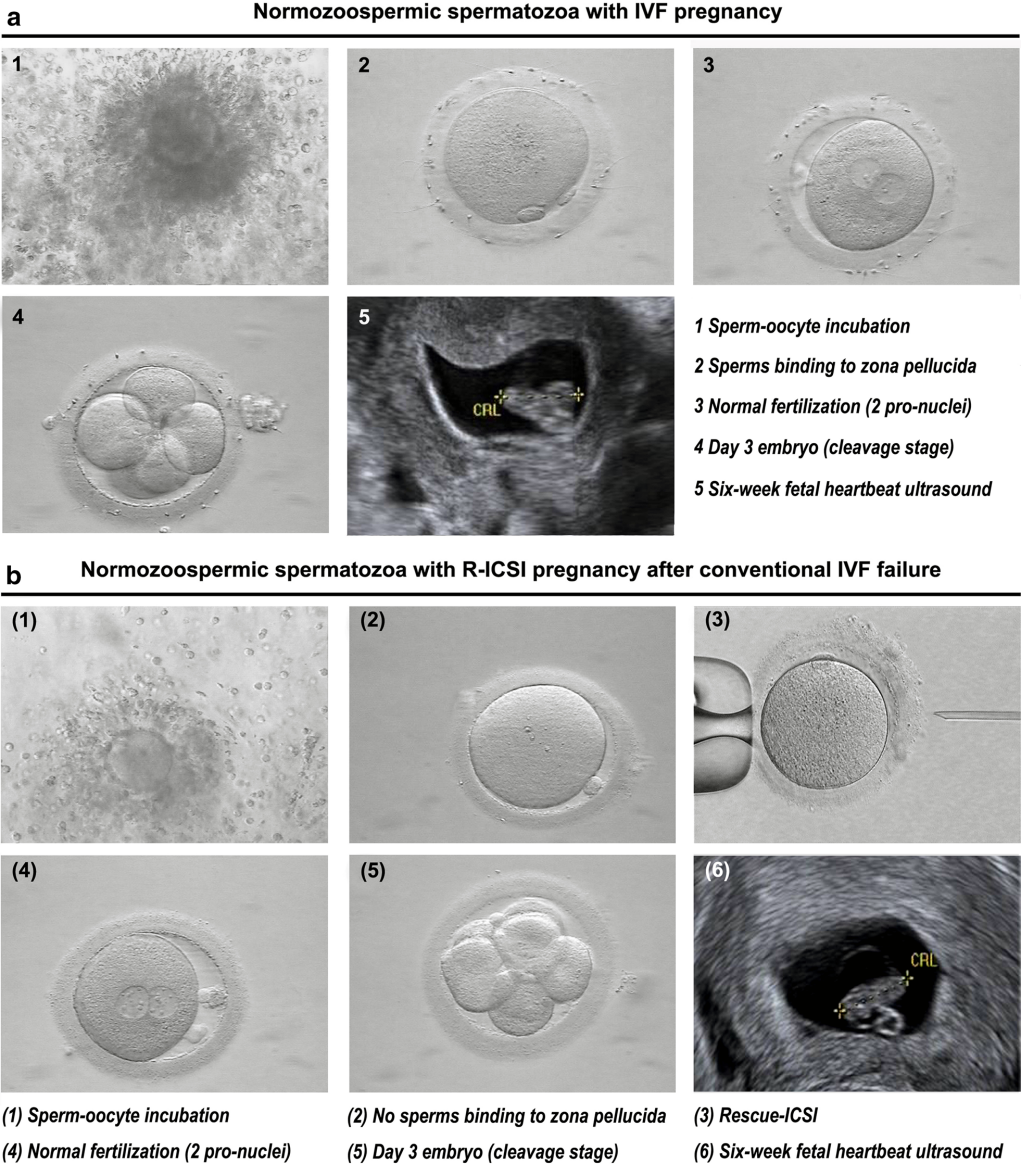
Representative normozoospermic spermatozoa with IVF pregnancy or R-ICSI pregnancy after conventional IVF treatment. **a1** The retrieved oocyte was incubated with the normal sperms, and the concentration of sperms was 300,000/mL; **a2** Two polar bodies were visible at 4 h after IVF; **a3** Two pronuclei were visible at 16 h after IVF, which was considered as normal fertilization; **a4** day 3 cleavage stage embryo; **a5** Clinical pregnancy was confirmed by ultrasonographic evidence of the fetal heartbeat at 6 weeks of gestation. **b1** The retrieved oocyte was incubated with the normal sperms, and the concentration of sperms was 300,000/mL; **b2** No second polar body was visible at 4 h after IVF; **b3** R-ICSI was performed at 5 h after IVF; **b4** Two pronuclei were visible at 16 h after IVF, which was considered as normal fertilization; **b5** day 3 cleavage stage embryo; **b6** Clinical pregnancy was confirmed by ultrasonographic evidence of the fetal heartbeat at 6 weeks of gestation

In accordance with World Health Organization (WHO) guidelines, all the men recruited in this study were normozoospermic. They had normal BMI, semen volume, total sperm count, progressive motility, sperm concentration, sperm morphology. Their partners were also characterized with normal BMI, FSH, LH, E2, number of retrieved oocytes, and without PCOS, endometriosis, endocrine disease or chromosomal abnormalities. The statistical analysis showed no differences of these reproductive parameters between the IVF pregnancy group and the R-ICSI pregnancy group (Table 1). Considering the subsequently successful clinical pregnancy and the phenotype with a dramatical decrease of sperm binding to the zona pellucida, male factor was the cause of failed fertilization at IVF, instead of their female partners (Fig. 1).

**Table 1:** Semen analysis and IVF cycle data (Mean ± SEM)

Characteristics	Normozoospermic men with IVF pregnancy^a^	Normozoospermic men with R-ICSI pregnancy^a^	*P* value
Semen			
Age	32.1 ± 0.2	31.6 ± 0.2	0.125
BMI (kg/m^2^)	22.6 ± 0.1	22.3 ± 0.1	0.161
Volume (mL)	2.9 ± 0.1	3.0 ± 0.1	0.410
Total sperm count (10^6^/ejaculate)	179.0 ± 3.2	187.4 ± 4.1	0.114
Progressive motility (%)	37.1 ± 0.3	37.7 ± 0.3	0.139
Sperm morphology (normal %)	7.1 ± 0.1	7.2 ± 0.2	0.555
IVF data			
Age	31.7 ± 0.2	31.6 ± 0.2	0.563
BMI (kg/m^2^)	21.9 ± 0.2	21.8 ± 0.2	0.757
Basal FSH (U/L)	6.3 ± 0.1	6.4 ± 0.1	0.179
Basal LH (U/L)	3.5 ± 0.1	3.3 ± 0.1	0.111
Basal E2 (ng/L)	32.4 ± 0.5	32.8 ± 0.4	0.487
No. of retrieved oocytes	13.2 ± 0.3	13.5 ± 0.3	0.501
No. of 2 pro-nuclear zygotes	9.2 ± 0.2	9.3 ± 0.2	0.658

^a^An ultrasound detection of fetal heartbeat 6 weeks after embryo

### Spontaneous acrosome reaction

**Fig. 2:**
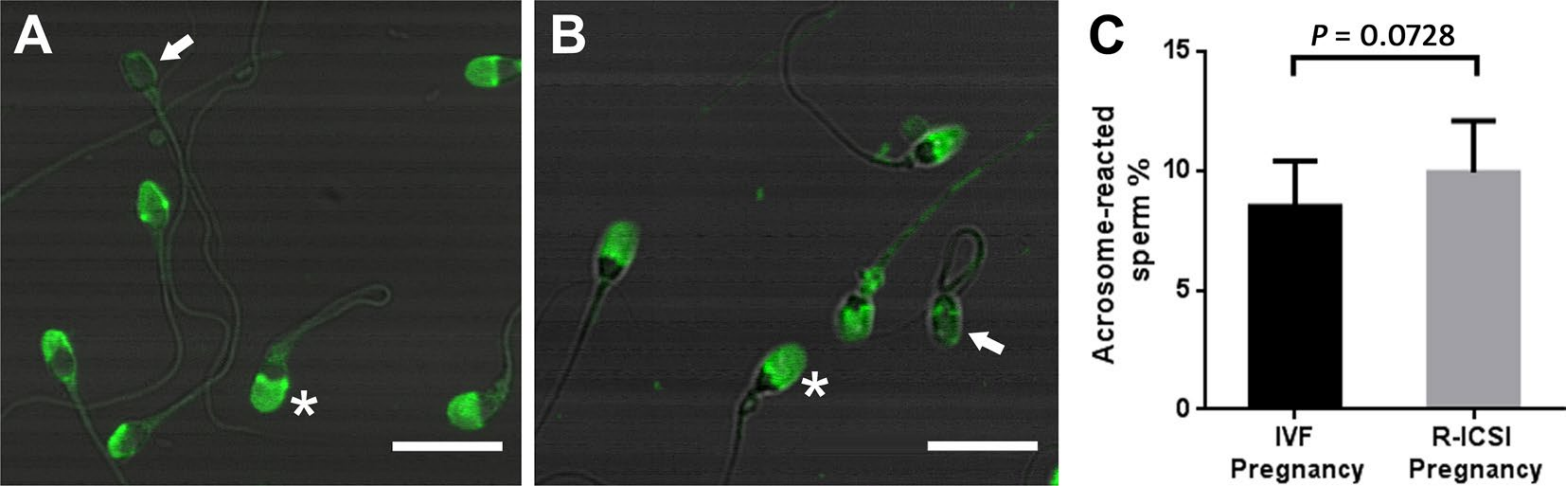
Representative fluorescence images and quantification of FITC-PSA staining. **A** The detection of spontaneous acrosome reaction in the IVF pregnancy group; **B** The detection of spontaneous acrosome reaction in the R-ICSI pregnancy group; **C** The quantification of spontaneous acrosome reaction between the IVF-pregnancy group (n = 20) and the R-ICSI pregnancy group (n = 20). Acrosome-intact sperm is marked by asterisk, and acrosome-reacted sperm is marked by arrow. Each bar indicates 10 μm

FITC-PSA was applied to determine the difference of the spontaneous acrosome reactions between the IVF pregnancy group and R-ICSI pregnancy group. In comparison with sperm from the normozoospermic men of IVF pregnancy group, the percentage of acrosome-reacted sperm showed a non-significant increase in normozoospermic men of R-ICSI group (*P *= 0.0756) (Fig. 2).

### Comparative analysis of normozoospermic sperm proteins between the IVF pregnancy group and the R-ICSI pregnancy group

**Fig. 3:**
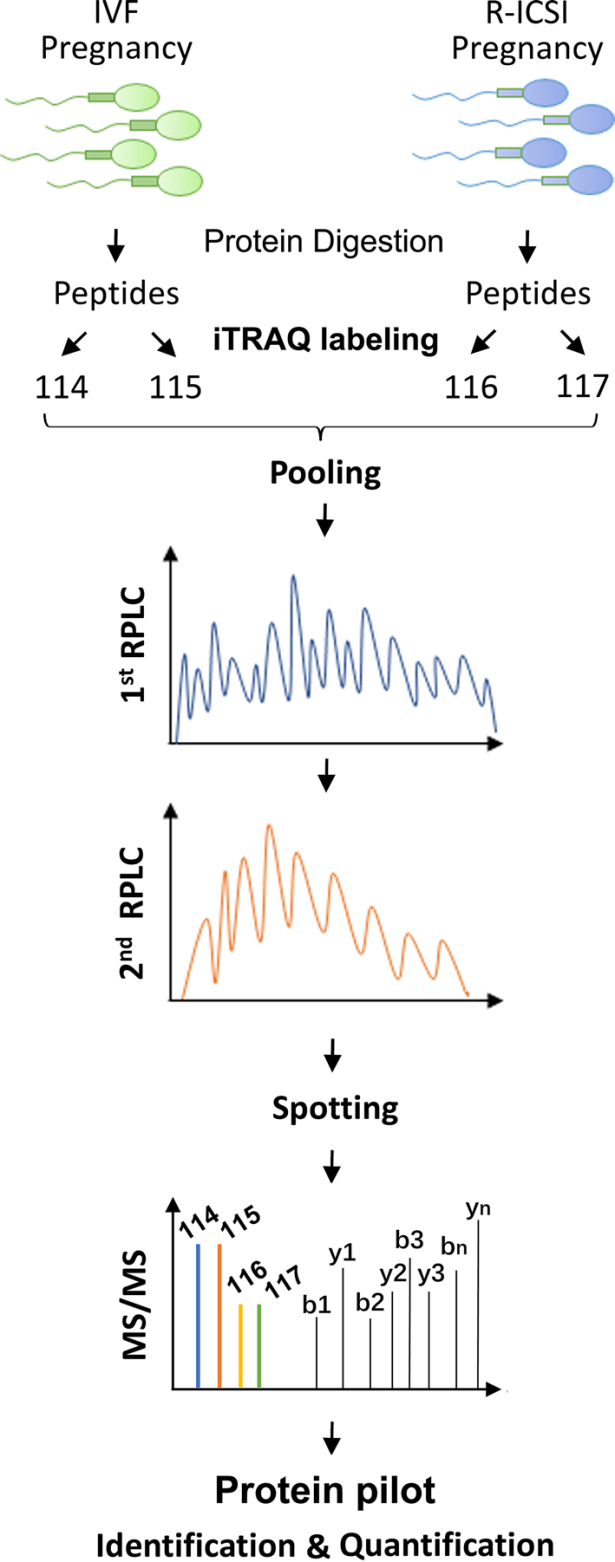
Experimental workflow of iTRAQ-based sperm proteome associated with different IVF outcomes. Equal amounts of sperm proteins from 20 normozoospermic men from IVF pregnancy group and 20 normozoospermic men from R-ICSI pregnancy group were pooled, digested by trypsin, and labelled by iTRAQ labels. After the labeled samples were separated by a pH10.0 reversed phase chromatography and a pH3.0 reversed phase chromatography, the fractions were spotted on the MALDI plates, and sequenced by the 5800 MALDI-TOF/TOF mass spectrometry. The identification and quantification of sperm proteins were performed by the software Protein pilot (version 4.0)

**Fig. 4:**
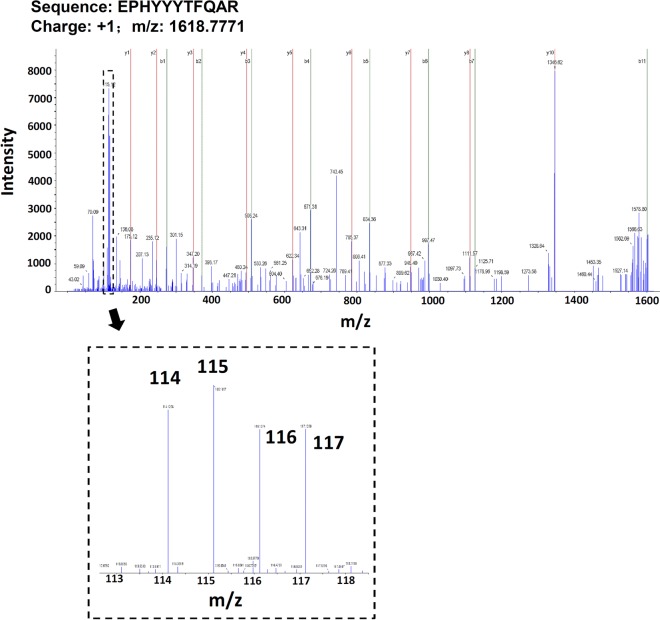
Representative spectrum of sperm protein sequenced by 5800 MALDI TOF/TOF mass spectrometry. The MS/MS map is identified as respectively for zona pellucida-binding protein 1 (ZPBP1). The sequence of precursor at m/z 1618.7771 is assigned to the peptide of EPHYYYTFQAR. The enlarged map represents the ions labelled by iTRAQ reagent

Figure 3 indicates the flowchart of iTRAQ-based proteomic analysis of normozoospermic sperm from IVF and R-ICSI pregnancy group. Briefly, the sperm proteins were digested by trypsin, labeled by iTRAQ reagents, separated by two-dimension RPLC, spotted on the MALDI plate, and sequenced by mass spectrometry. The ProteinPilot™ software was applied to analyze the spectraby searching the reviewed Swiss-Prot human database (20,316 sequences, 2018_02 released). A total of 450 sperm proteins were identified and quantified with a high confidence (FDR < 0.01) (Additional file 1: Supplementary Table 1). Compared with the IVF pregnancy group, 36 sperm proteins were up-regulated (ratio_R-ICSI/IVF_ > 1.274, *P *< 0.05) (Table 2), and 20 sperm proteins were down-regulated (ratio_R-ICSI/IVF_ < 0.762, *P *< 0.05) (Table 3) in the R-ICSI pregnancy group. Figure 4 shows the representative MS/MS spectrum of iTRAQ-labelled peptide from zona pellucida-binding protein 1 noted with most b-ions and y-ions.

**Table 2:** Higher abundant sperm proteins in normozoospermic samples that achieved pregnancy undergoing R-ICSI after IVF failure (n = 36)

Swiss-Prot accession number	Protein name	Ratio (Mean ± SD) R-ICSI pregnancy/IVF pregnancy
P62805	Histone H4	2.62 ± 0.03
O95996	Adenomatous polyposis coli protein 2	2.29 ± 0.12
Q8NCQ7	Protein PROCA1	2.13 ± 0.12
P04554	Protamine-2	1.97 ± 0.12
P19652	Alpha-1-acid glycoprotein 2	1.93 ± 0.38
P02766	Transthyretin	1.77 ± 0.10
P63167	Dynein light chain 1	1.72 ± 0.09
Q13200	26S proteasome non-ATPase regulatory subunit 2	1.66 ± 0.23
P02749	Beta-2-glycoprotein 1	1.57 ± 0.11
P31937	3-hydroxyisobutyrate dehydrogenase	1.56 ± 0.01
O14556	Glyceraldehyde-3-phosphate dehydrogenase, testis-specific	1.55 ± 0.02
P02763	Alpha-1-acid glycoprotein 1	1.54 ± 0.21
P02760	Protein AMBP	1.53 ± 0.04
P55064	Aquaporin-5	1.53 ± 0.11
Q9Y4G6	Talin-2	1.52 ± 0.20
P38567	Hyaluronidase PH-20	1.51 ± 0.07
Q6UWM5	GLIPR1-like protein 1	1.49 ± 0.13
P25786	Proteasome subunit alpha type-1	1.48 ± 0.11
Q9BYD9	Actin-related protein T3	1.48 ± 0.16
P02765	Alpha-2-HS-glycoprotein	1.47 ± 0.07
P04217	Alpha-1B-glycoprotein	1.45 ± 0.10
P02790	Hemopexin	1.44 ± 0.08
P25311	Zinc-alpha-2-glycoprotein	1.44 ± 0.08
P49748	Very long-chain specific acyl-CoA dehydrogenase	1.43 ± 0.08
Q68DN1	Uncharacterized protein C2orf16	1.42 ± 0.13
Q9BZW7	Testis-specific gene 10 protein	1.40 ± 0.03
Q96FJ2	Dynein light chain 2	1.39 ± 0.03
Q71DI3	Histone H3.2	1.39 ± 0.10
A6NMS7	Leucine-rich repeat-containing protein 37A	1.39 ± 0.07
P43652	Afamin	1.32 ± 0.03
Q8N0W7	Fragile X mental retardation 1 neighbor protein	1.32 ± 0.14
P49721	Proteasome subunit beta type-2	1.31 ± 0.07
Q5VZ72	Izumo sperm-egg fusion protein 3	1.30 ± 0.13
Q96QV6	Histone H2A type 1-A	1.29 ± 0.22
Q96KR1	Zinc finger RNA-binding protein	1.29 ± 0.10
P38117	Electron transfer flavoprotein subunit beta	1.28 ± 0.12

Annotations of altered proteins identified in sperm samples from the R-ICSI pregnancy group compared with the IVF pregnancy group. Values > 1.274 (*P *< 0.05) correspond to higher abundance in the R-ICSI pregnancy group. Standard deviation (SD)

**Table 3:** Lower abundant sperm proteins in normozoospermic samples that achieved pregnancy undergoing R-ICSI after IVF failure (n = 20)

Swiss-Prot accession number	Protein name	Ratio (Mean ± SD) R-ICSI pregnancy/IVF pregnancy
P62937	Peptidyl-prolyl cis–trans isomerase A	0.76 ± 0.01
Q5VTE0	Putative elongation factor 1-alpha-like 3	0.76 ± 0.07
Q9BXU7	Ubiquitin carboxyl-terminal hydrolase 26	0.76 ± 0.08
Q9BS86	Zona pellucida-binding protein 1	0.75 ± 0.02
P02743	Serum amyloid P-component	0.75 ± 0.02
Q9UKU0	Long-chain-fatty-acid–CoA ligase 6	0.75 ± 0.16
Q8N4L4	Spermatid maturation protein 1	0.74 ± 0.03
Q8NEB7	Acrosin-binding protein	0.74 ± 0.09
Q13748	Tubulin alpha-3C/D chain	0.74 ± 0.04
P30101	Protein disulfide-isomerase A3	0.74 ± 0.05
P69905	Hemoglobin subunit alpha	0.72 ± 0.03
Q1ZYL8	Izumo sperm-egg fusion protein 4	0.71 ± 0.01
Q6NXR0	Interferon-inducible GTPase 5	0.70 ± 0.02
P07477	Trypsin-1	0.70 ± 0.10
P84085	ADP-ribosylation factor 5	0.68 ± 0.13
Q13642	Four and a half LIM domains protein 1	0.67 ± 0.05
Q499Z3	Schlafen-like protein 1	0.61 ± 0.06
P00352	Retinal dehydrogenase 1	0.61 ± 0.11
Q06830	Peroxiredoxin-1	0.60 ± 0.12
Q96C74	Ropporin-1-like protein	0.55 ± 0.07

Annotations of altered proteins identified in sperm samples from the R-ICSI pregnancy group compared with the IVF pregnancy group. Values < 0.762 (*P *< 0.05) correspond to lower abundance in the R-ICSI pregnancy group. Standard deviation (SD)

### GO classification of altered sperm proteins associated with R-ICSI pregnancy after conventional failed IVF

**Fig. 5:**
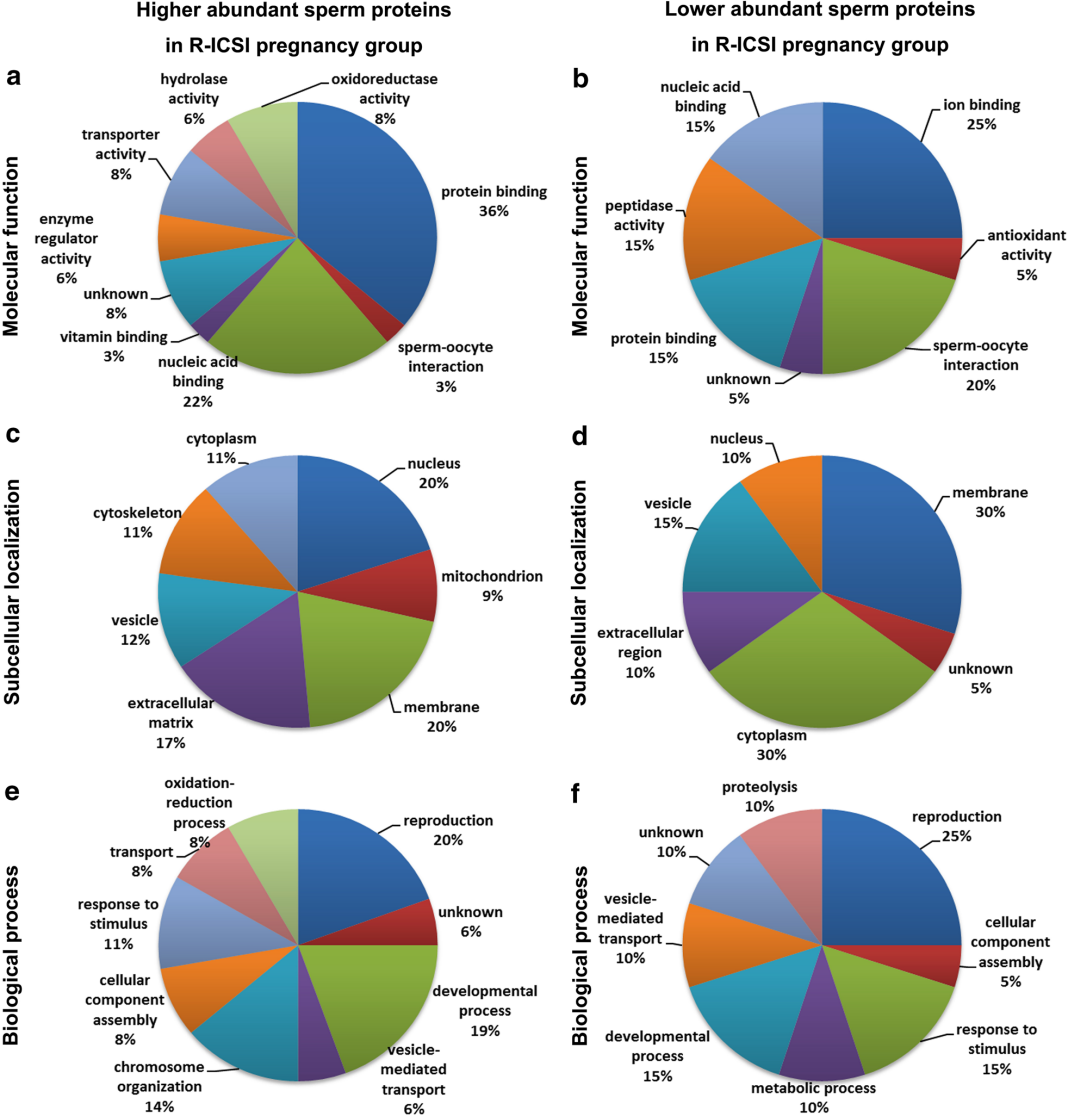
Pie diagrams of the R-ICSI pregnancy-related sperm proteins categorized by GO classifications. **a**, **c** and **e** represent the higher abundant proteins in the R-ICSI group, and **b**, **d** and **f** represent the lower abundant proteins in the R-ICSI group. **a**, **b** are molecular function, **c**, **d** are subcellular localization, and **e**, **f** are biological process

Based on PANTHER, DAVID and the PubMed literature, the differentially expressed sperm proteins were classified into GO categories (Fig. 5). As for higher abundant sperm proteins in the R-ICSI pregnancy group, protein binding (36%) and nucleic acid binding (22%) were the main molecular functions; nucleus (20%) and membrane (20%) were the majority of subcellular localizations; reproduction (20%), developmental process (19%), chromosome organization (14%) were the important biological processed. In the lower abundant sperm proteins in the R-ICSI pregnancy group, sperm-oocyte interaction (20%) was the main molecular function, membrane (30%) accounted for a large portion of subcellular localization, and reproduction (25%) and development process (15%) were the main biological processed.

### Interaction Networks of sperm proteins involved in the R-ICSI pregnancy after conventional IVF failure

**Fig. 6:**
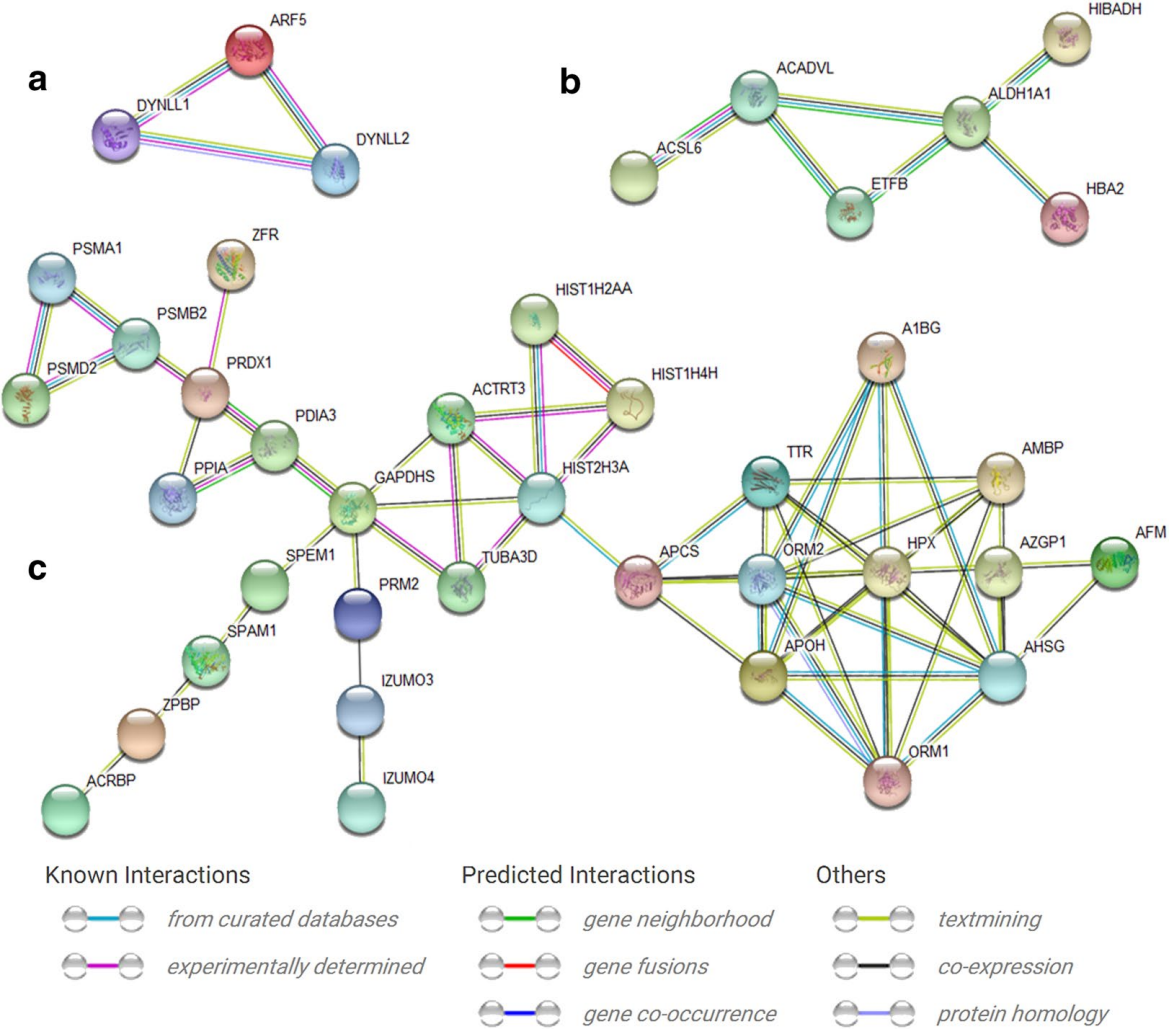
Representative protein–protein interaction networks of altered R-ICSI pregnancy-related sperm proteins. A total of 40 genes was classified into 3 clusters. **a** Cluster contained three nodes, **b** cluster included six nodes, and **c** cluster was consisted of 31 nodes. The relationships were derived from the curated databases, experimentally determined, gene neighborhood, gene fusions, gene co-occurrence, textmining, co-expression, and protein homology

The online STRING software was applied to construct the protein–protein interaction network of the altered sperm proteins related to the R-ICSI pregnancy after failed IVF treatment (Fig. 6). Forty differentially expressed proteins were classified into three interaction groups. First group included 3 sperm proteins (ADP-ribosylation factor 5, dynein light chain 1, and dynein light chain 2), second group consisted of 6 sperm proteins (long chain fatty acid CoA ligase 6, hemoglobin subunit alpha, retinal dehydrogenase 1, very long chain specific acyl CoA dehydrogenase, electron transfer flavoprotein subunit beta, and 3-hydroxyisobutyrate dehydrogenase), and third group covered the left31 proteins (zona pellucida-binding protein 1, acrosin-binding protein, spermatid maturation protein 1, izumo sperm-egg fusion protein 4, tubulin alpha-3C/D chain, peroxiredoxin-1, peptidyl-prolyl cis–trans isomerase A, protein disulfide-isomerase A3, serum amyloid P-component, actin-related protein T3, histone H2A type 1-A, zinc finger RNA-binding protein, histone H3.2, izumo sperm-egg fusion protein 3, 26S proteasome non-ATPase regulatory subunit 2, histone H4, proteasome subunit beta type-2, afamin, hyaluronidase PH-20, proteasome subunit alpha type-1, zinc-alpha-2-glycoprotein, alpha-1-acid glycoprotein 2, protamine-2, alpha-1B-glycoprotein, hemopexin, transthyretin, alpha-2-HS-glycoprotein, alpha-1-acid glycoprotein 1, protein AMBP, beta-2-glycoprotein 1, glyceraldehyde-3-phosphate dehydrogenase).

### Comparison of present sperm proteome and the previous reported IVF-related sperm proteomes

**Fig. 7:**
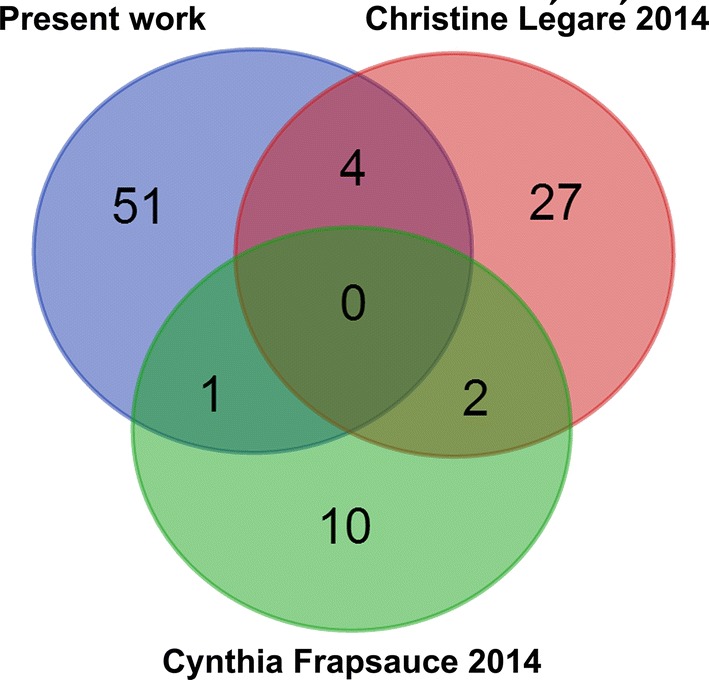
Venn diagram of the overlaps of the present work and the other two differential IVF-related sperm proteomes (Légaré et al. [8], Frapsauce et al. [7])

To indicate the overlap of the IVF-related sperm proteomes between different labs and to explore the new findings, the previous published sperm profiles associated with the different outcome under the IVF treatment were selected (7, 8), and Fig. 7 shows the overlap between them. Compared with the present work, approximate 90% IVF-related sperm proteins were not identified in the previous researches.

### Western blot analysis

**Fig. 8:**
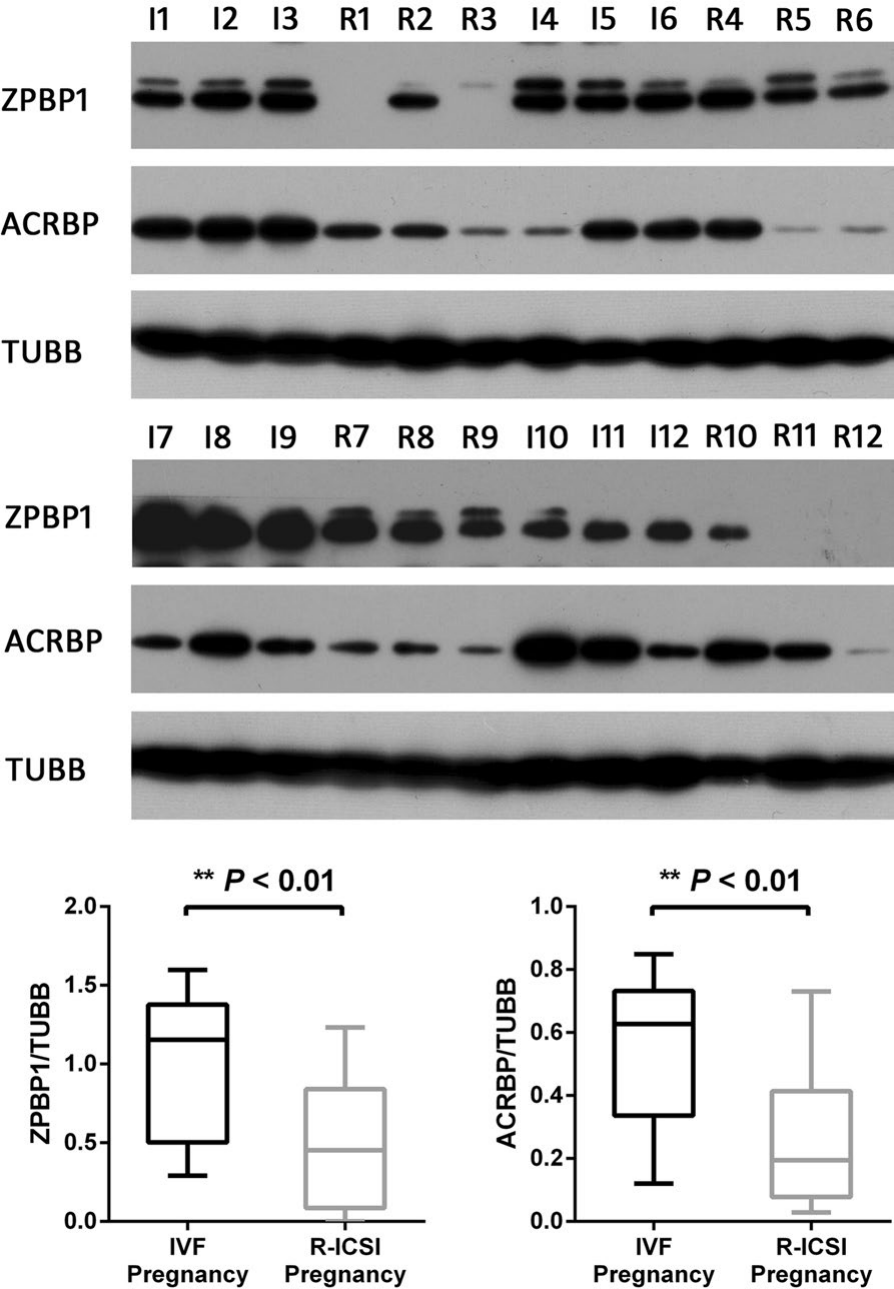
Western blot analyses of zona pellucida-binding protein 1 (ZPBP1) and acrosin-binding protein (ACRBP). The expressions of ZPBP1 and ACRBP were analyzed with the individual samples (the IVF pregnancy group (I1-I12); the R-ICSI pregnancy group (R1-R12)). Asterisk denotes a statistically difference between the two groups

To validate theconfidence of iTRAQ technology for protein quantification, western blot was used to analyze the expression patterns of zona pellucida-binding protein 1 (ZPBP1) and acrosin-binding protein (ACRBP) by the individual samples (Fig. 8). The outcome indicated the levels of ZPBP1 and ACRBP were significantly lower in the R-ICSI pregnancy group than in the IVF pregnancy group (*P* value < 0.01), which were consistent with the proteomic results.

### Immunofluorescence quantification

**Fig. 9:**
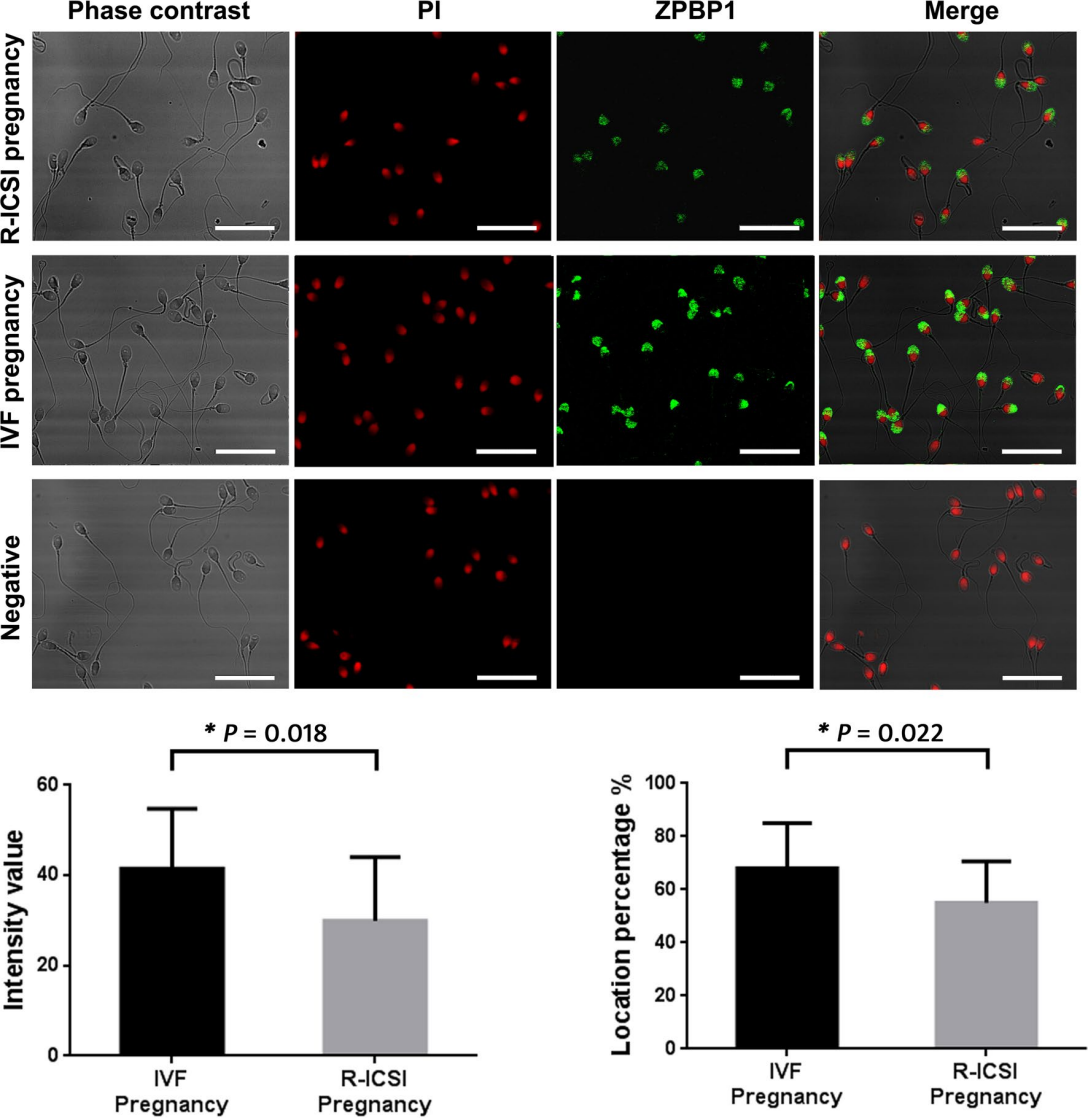
Quantitative immunofluorescence of zona pellucida-binding protein 1 (ZPBP1) on the spermatozoa samples from the IVF pregnancy group (n = 20) and the R-ICSI pregnancy group (n = 20). IgG was used as negative control. PI, propidium iodide. Scale bar indicates 20 μm. Asterisk indicates significant difference analyzed by unpaired t-test (*P *< 0.05)

Further immunofluorescence analysis demonstrated that zona pellucida-binding protein 1 was located on the acrosome region of spermatozoa from IVF pregnancy group and R-ICSI pregnancy group. But the percent of spermatozoa with positive staining and fluorescence intensity were statistically decreased in normozoospermic men with R-ICSI pregnancy compared to normozoospermic men with IVF pregnancy (*P *< 0.05, Fig. 9).

## Discussion

Hitherto, there is still unclear about the underlying mechanisms of the unexplained infertility in the normozoospermic men. In the R-ICSI group, although the parameters of retrieved oocytes were normal, there was still a lack of sperm fixation to the zona pellucida. Meanwhile, to exclude the infertile factors from their female partners as far as possible, the subsequent pregnancies were all confirmed by an ultrasound detection of 6-week fetal heartbeat after fertilization by IVF or R-ICSI treatment in this study. Therefore, we provided a hypothesis of the sperm-oocyte interaction molecule defects underlying this unexplained male factor infertility.

To discover the critical infertile molecules, the proteomic technique including two-dimensional electrophoresis has been widely used to realize these goals [6–8]. Compared with the previous conventional proteomic researches, the powerful iTRAQ based quantitative proteomics was applied to reveal more altered sperm proteins in this work. The overlap of the IVF-related sperm proteomes indicated only 5 of 56 differential proteins have been identified and quantified such as protein disulfide-isomerase A3, histone H3.2, histone H4, glyceraldehyde-3-phosphate dehydrogenase, and tubulin alpha-3C/D chain in the overlap. The low common identification of sperm proteins might be mainly caused by different experimental design, patient recruitment, sperm handling, quantitative methods, and data processing. in the future, the collaboration of international laboratories should be established to provide the better understanding on male infertility.

Through the analysis of the online STRING software three protein–protein interaction networks were delineated with 40 altered sperm proteins involved in R-ICSI pregnancy. The biggest interaction network contained 31 nodes including several down-regulated proteins associated with sperm-oocyte interaction, e.g. acrosin-binding protein (ACRBP) and zona pellucida-binding protein 1 (ZPBP1), which displayed the complexity of the mechanisms under the male infertility. ACRBP is a proacrosin binding protein and participate in the process of the acrosin zymogen condensation [26], and ZPBP1 also plays an important role in acrosome compaction [27]. ACRBP and ZPBP1 has been identified as high molecular weight complexes from sperm head plasma membrane and defined as the acrosomal proteins. As the associated partner of proacrosin (with zona pellucida affinity), the transportation of ACRBP from acrosome to sperm head surface seems to be involved in the initial binding between sperm head surface and zona pellucida, which subsequently induce the acrosome reaction. During the secondary sperm binding, the exposed inner acrosomal membranes of the acrosome-reacted sperm further fix to and penetrate the zona pellucida [28]. ZPBP1 isa acrosomal protein with the zona pellucida glycoprotein affinity, which locates on the inner acrosomal membrane of the anterior acrosome, and the inner acrosomal membrane and the outer acrosomal membrane of the equatorial segment. Even after the acrosome reaction, ZPBP1 is still located on the inner acrosomal membrane, which facilitates the secondary sperm binding to zona pellucida and the following zona penetration [29, 30]. Another research has reported that ZPBP1 competes with proacrosin (the binding substrate of ACRBP) for fixation to the zona pellucida during the fertilization process [31]. Recent study indicated ZPBP1 gene mutation is also involved in the teratospermia [32]. In the present work, ACRBP and ZPBP1 were both statistically down-regulated in the R-ICSI pregnancy group after the failure of conventional IVF. Lower ACRBP expression might not only weaken the initial binding to zona pellucida but also increase the level of free proacrosin. The latter would compete with ZPBP1 for binding to zona pellucida. In addition, the decreased ZPBP1 also could attenuate the secondary sperm binding to zona pellucida. As a result, the failed insemination characterized with no binding to the zona pellucida could appear in the previously clinical IVF treatment.

In addition to the above altered sperm proteins, a dramatical decrease of sperm binding to the zona pellucida also indicated that there might be some defects on the acrosome reaction in the R-ICSI pregnancy group. Acrosome is an important organelle for the fertilization. The anterior acrosome was related to sperm-zona pellucida interaction, and the posterior acrosome is associated with sperm-egg fusion [33]. The occurrence of sperm acrosome reaction releases some necessary lysins, which facilitates sperm to penetrate the zona pellucida and fuse with oocyte membrane [34, 35]. That is to say, acrosome reaction should occur after the sperm binding to the zona pellucida. Otherwise, the spontaneous occurrence of acrosome reaction without intact with the zona pellucida will deprive the sperm fertilization potential [36]. But spontaneous acrosome reaction can arise before contact with the zona pellucida, which is associated with the low reproductive potential. It has been reported that the spontaneous acrosome reaction rate is higher in the spermatozoa from obese group than from lean group [37]. Obesity is usually related to a higher risk for male subfertility, which is characterized with the reduced sperm concentration, count and motility [38]. In 1984, Plachot et al. suggested no correlation could be found between the acrosomal reaction rate and the ability to fertilize the oocyte [39]. Recently, spontaneous acrosome reaction has been used to evaluate the semen quality during IVF, which provides a new way to decide whether conventional insemination or ICSI is preferable treatment. As a result, the samples with a high rate of spontaneous acrosome reaction shows a significant low fertilization rate compared with those with a low rate of spontaneous acrosome reaction [40]. In the present work, the BMI index of both IVF-pregnancy group and R-ICSI pregnancy group were normal, and without statistical difference. Therefore, obesity is not cause of the occurrence of spontaneous acrosome reaction. This unexplained higher spontaneous acrosome reaction rate might also be responsible for the failure of conventional insemination.

## Conclusions

In the past decades, IVF has been widely applied to help the couples undergoing the failed natural fertilization. To explore the male infertile molecular causes of the failed IVF (normozoospermic men with a severe decrease of sperm binding to the zona pellucida of the normal oocytes), an iTRAQ proteomic approach was used to reveal the infertility-related differential sperm proteins between the R-ICSI pregnancy group and the conventional IVF pregnancy group men. The new panel of the altered sperm proteins, especially these sperm-oocyte interaction proteins will provide novel clues to understand the mechanisms of male infertility and become the potential biomarkers for the better prediction of IVF outcomes. Further studies are still necessary to confirm the feasibility of these potential markers used for the diagnosis and prognosis of male infertility.

## Additional file

**Additional file 1: Supplementary Table 1.** The quantitative details of sperm proteins from normozoospermic men between the IVF pregnancy group and the R-ICSI pregnancy group.

## Abbreviations

IVF: in vitro fertilization
R-ICSI: rescue intracytoplasmic sperm injection
iTRAQ: isobaric tags for relative and absolute quantification
RPLC: high-performance liquid chromatography
MALDI: matrix-assisted laser desorption/ionization
MS: mass spectrometry
WHO: world health organization
PCOS: polycystic ovary syndrome
FITC: fluorescein isothiocyanate
PSA: pisum sativum agglutinin
PANTHER: protein analysis through evolutionary relationships
DAVID: the database for annotation, visualization and integrated discovery
STRING: search tool for recurring instances of neighbouring genes
BMI: body mass index
FSH: follicle-stimulating hormone
LH: luteinizing hormone
E2: estradiol
FDR: false discovery rate
ACRBP: acrosin-binding protein
ZPBP1: zona pellucida-binding protein 1
